# A Fixed, Unreducible, Unstable Medial Swivel Dislocation of the Talonavicular Joint with Associated Navicular Fracture

**DOI:** 10.1155/2021/9959830

**Published:** 2021-06-14

**Authors:** James T. Layson, Alan Afsari, Todd Peterson, David Knesek, Benjamin Best

**Affiliations:** ^1^Ascension Macomb-Oakland, 11800 Twelve Mile Rd, Warren, MI 48093, USA; ^2^Ascension St. John Hospital, 22101 Moross Road, Suite #214 Detroit, MI 48236, USA; ^3^Capital Orthopaedics and Sports Medicine, 12499 University Ave., Suite #210 Clive, IA 50325, USA; ^4^Motor City Orthopedics and Ascension Providence Hospital, 26850 Providence Parkway, #260 Novi, MI 48374, USA; ^5^Ascension Macomb-Oakland and Ascension St. John Hospital, 22101 Moross Road, Suite #214 Detroit, MI 48236, USA

## Abstract

A 32-year-old white male was on a second-story balcony when he fell off and landed on the cement below. With initial X-rays being read as negative on the radiology report due to the subtle nature of the injury, the patient was promptly diagnosed with a medial swivel dislocation by the orthopaedic team, which ended up being fixed, unstable, and irreducible. The patient also had acute skin compromise and needed to be taken to the operating room prior to progression of skin breakdown. This dislocation pattern is a rare variant, especially when paired with the fixed nature of the dislocation and the soft tissue compromise. In the end, open treatment was necessary in order to reduce the talonavicular joint. Because of early recognition and prompt treatment, skin breakdown was avoided. Internal screw fixation of the fractured navicular bone was needed along with K-wire insertion to hold the normal anatomy of the talonavicular joint reduced. All hardware was ultimately removed after healing, and anatomy was restored with excellent patient function. This case report highlights the orthopaedic knowledge needed to not only recognize this rare fracture-dislocation pattern but to also treat it promptly when encountered.

## 1. Introduction

In 1975, Main and Jowett first described a variant of the subtalar dislocation which they termed a “swivel dislocation” of the midtarsal joint. This was described as a navicular body fracture, either isolated or with additional fractures, dislocations, and/or fracture dislocations of the foot [1]. The medial swivel dislocation is a rare injury in the literature [2]. This is the only case we have found that reported of a “fixed' or “irreducible” medial swivel dislocation with acute soft tissue compromise.

Importantly, swivel dislocations can easily be missed due to the subtle clinical and radiographic findings [3, 4]. Miller et al., Richter et al., and Ip and Lui have all advocated the importance of internal fixation in these injuries [5–8]. Due to the soft tissue compromise, urgent understanding is critical. This case report is unique because of the surgical strategies employed to open reduce and stabilize this irreducible medial swivel joint and talonavicular dislocation, ultimately avoiding soft tissue compromise.

## 2. Case Presentation

A 32-year-old male was working on a second story balcony when he fell off and landed on the cement below. He was transported to our level one trauma center emergency department where he complained of left foot pain. The emergency room reported negative foot and ankle X-rays and diagnosed the patient with an ankle sprain. The radiology report of the foot was “negative for fracture or dislocation.”

Orthopaedic surgery was consulted due to the patient's inability to walk. During evaluation, physical exam of his foot revealed a subtle sulcus sign present over the talonavicular joint and significant dorsal soft tissue pressure over the navicular bone. There was full sensation and motor function of his extremity. He had a palpable dorsalis pedis and posterior tibial artery.

**Figure 1:**
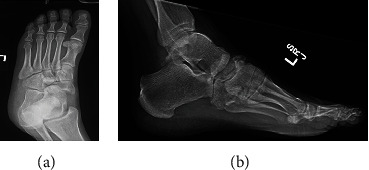
(a) Initial AP X-ray of the left foot demonstrating subtle talonavicular dislocation, read as negative by radiology. (b) Initial lateral X-ray of the left foot demonstrating subtle talonavicular dislocation, read as negative by radiology.

Initial plain X-rays of the foot evaluated by orthopaedics demonstrated a navicular fracture with a medial dislocation of the navicular on the talus (Figures 1(a) and 1(b)). A cuboid fracture and a fracture of the proximal metaphysis of the third metatarsal were also identified, along with a small fracture of the lateral aspect of the anterior process of the calcaneus.

**Figure 2:**
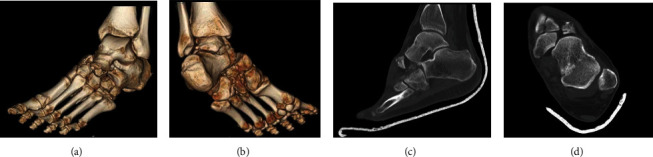
(a) 3D CT scan demonstrating swivel injury, comminuted fracture of the navicular and cuboid bone, and calcaneocuboid joint subluxation. (b) 3D CT scan from medial viewpoint showing the medial dislocation of the talonavicular joint. (c) Sagittal CT cut of the left foot showing both talonavicular and subtle calcaneocuboid joint subluxations. (d) Axial cut of the CT scan showing medial dislocation of the talonavicular joint left foot; note the navicular fracture fragment that remained in the anatomic position; this “constant fragment” received screw fixation to aid in reconstruction of the navicular bone.

A computerized tomography (CT) scan was ordered by orthopaedic surgery, confirming a comminuted lateral navicular fracture with navicular medial dislocation on the talar. An impacted fracture of the anterior calcaneal process was also seen with a comminuted cuboid fracture and a third metatarsal fracture (Figure 2). In addition, the calcaneocuboid and subtalar joints were subluxed (Figures 2(a) and 2(c)). The patient was then placed in a short leg posterior splint for stabilization. Closed reduction of this fixed injury was not possible under general sedation in the emergency room. Due to this, the decision was made to take the patient to the operative suite for open talonavicular joint reduction and possible open reduction and internal fixation of navicular, cuboid, calcaneus, and Chopart's joint.

**Figure 3:**
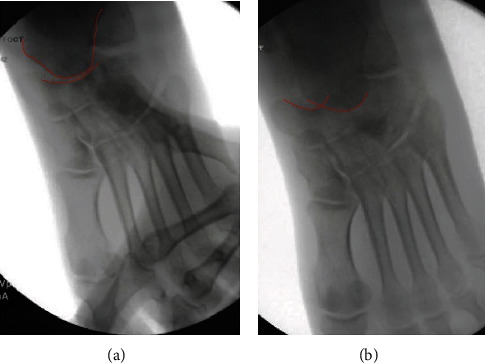
(a) Intraoperative image showing reduction after open treatment and direct pressure on the navicular bone to hold it reduced; the red line outlines both the navicular and talus bones at the talonavicular joint (TN joint is reduced). (b) Intraoperative image showing the talonavicular joint again with new dislocation after letting pressure off of the foot, demonstrating the unstable nature of the injury; the red line outlines both the navicular and talus bones at the talonavicular joint (TN joint is unreduced).

Open reduction of the talonavicular joint was performed first. The surgical incision was vertical in nature directly over the dorsal aspect of the talonavicular joint. We avoided the tibialis anterior and extensor hallucis longus tendons as well as the underlying neurovascular structures. The anterior capsule was torn, and open access to the joint achieved. Reduction was aided with direct visualization of the talonavicular joint, and direct pressure on the navicular bone with forefoot abduction achieved the final reduction (Figure 3(a)). However, as soon as forefoot abduction pressure was released, the joint would redislocate (Figure 3(b)).

**Figure 4:**
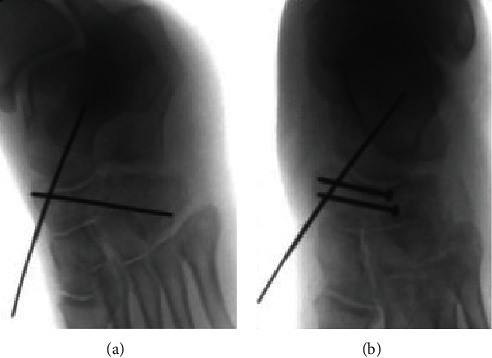
(a, b) Intraoperative images demonstrating the insertion of K-wires to hold the talonavicular joint reduction and addition of 2.7 mm screws in the “constant fragment” in order to reconstruct the anatomy of the navicular; the K-wires help to keep dislocation forces off of the screw fixation to avoid failure of the navicular screw fixation.

**Figure 5:**
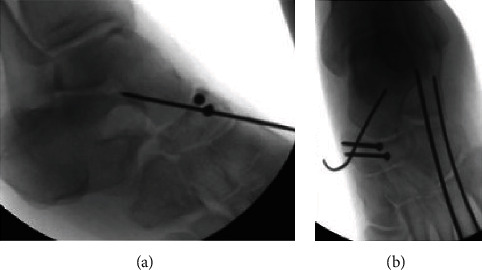
(a, b) Intraoperative images demonstrating the insertion of K-wires to hold the talonavicular joint reduction and addition of 2.7 mm screws in the “constant fragment” in order to reconstruct the anatomy of the navicular; the K-wires help to keep dislocation forces off of the screw fixation to avoid failure of the navicular screw fixation.

Due to this instability, K-wire stabilization was used to hold the navicular fracture and talotarsal reduction (Figures 4(a)and 5(b)). Navicular fracture reduction was found to stabilize Chopart's joint. Fixation was then performed with two fully threaded 2.7 mm screws to aid in navicular reduction (Figures 4(b) and 5(a)). The foot was then stressed, and Chopart's joint was found to be stable after this fixation. The cuboid and calcaneus fractures were treated closed without internal fixation. The patient was placed in a bulky Jones splint and instructed to be non-weight-bearing.

Screws were avoided in this case because they typically require removal at a later date, ultimately leading to another trip to the operating room. Temporary K-wire fixation allows for the potential for a single surgery to be performed and for the possibility to remove the pins in outpatient clinic. In this specific case, the K-wires happened to be removed in the operating room; however, no incision or dissection was needed, limiting further soft tissue trauma. It is our preference to use temporary K-wire fixation, and we will continue to use this for future cases with the plan for outpatient pin removal.

The patient was seen at two weeks and six weeks after the procedure, and the fracture and joint reductions remained intact. In our case, the pins were removed at 12 weeks to allow for optimal time for bony and ligamentous healing. While the pins could have potentially been removed sooner, we opted for a more conservative approach due to the nature of the rigidity of the involved joints, not typically requiring early range of motion. There is an inherent inflexible nature of the medial column of the foot, including the talonavicular joint. With this in mind, there was little benefit to early pin removal and motion, with obvious healing advantages if left in place for a longer period of time.

**Figure 6:**
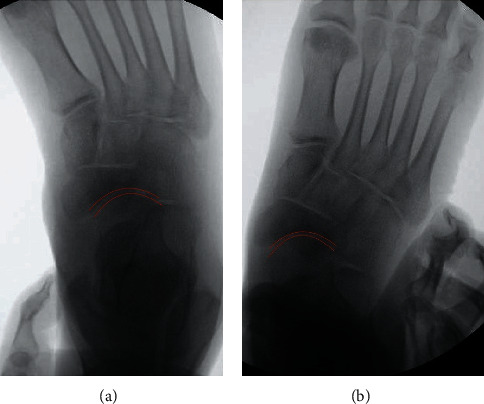
(a) 12-week follow-up with adduction stress of the forefoot. Intraoperative images of the foot showing normal navicular and talonavicular joint radiography and stability with real-time stress radiographs after complete healing and hardware removal; red lines demonstrate the joint surface of the talus and navicular at the talonavicular joint. (b) 12-week follow-up without adduction stress of the forefoot. Intraoperative images of the foot showing normal navicular and talonavicular joint radiography and stability with real-time stress radiographs after complete healing and hardware removal; red lines demonstrate the joint surface of the talus and navicular at the talonavicular joint.

He was scheduled for surgical hardware removal 12 weeks after his original injury, where the 3 K-wires and two navicular screws were removed. His foot was stressed with forefoot abduction and adduction under fluoroscopy and was found to remain stable. Stability was based on the findings that there was no incongruity when compared with the opposite foot during intraoperative stress radiography (Figures 6(a) and 6(b)).

He followed up again two weeks after his hardware removal and was advanced to weight-bearing as tolerated. Physical therapy was initiated, which included both ankle and subtalar range of motion exercises. This was done for a total of six weeks, and after completion, the patient regained nearly full range of motion in foot inversion, eversion, flexion, and extension. He declined follow-up at 5 years postoperatively but stated he was having no issues with function of his foot.

## 3. Discussion

It is important to be able to recognize the medial or lateral swivel dislocation, as the reduction maneuvers are different. Subtle X-ray findings related to this injury lead to a high rate of missed diagnosis in this particular injury. Our particular case was rare and peculiar because of the fixed nature of the dislocation. A combination of imaging (X-ray and CT scan) and physical exam must be used to aid in the diagnosis. These fracture dislocations are typically identified by dislocation of the talar head and navicular and typically include a navicular fracture. These dislocations are reduced by knee flexion (to relax the Achilles tendon musculature), accentuation of the deformity, and reversal of the deformity, sometimes being aided by direct digital pressure on the head of the talus.

Main and Jowett described the medial swivel dislocation as occurring through a medially directed force that dislocates the talonavicular joint, leaving the calcaneocuboid joint intact, but subluxing the subtalar joint [1]. The calcaneus is not totally displaced from the talus but rather rotates on the interosseous talocalcaneal ligament [5]. On the other hand, a lateral swivel dislocation occurs from a laterally directed force on the forefoot causing a lateral dislocation of the talonavicular joint and commonly a fracture of the cuboid (“nut cracker injuries”). In both of these “subtalar dislocation variants,” the talus remains positioned under the tibia [5].

A swivel dislocation is typically reduced by digital pressure on the talar head accompanied with adduction (lateral swivel injury) or abduction (medial swivel injury) of the forefoot with needed dorsiflexion or plantarflexion [2, 9]. This case is unique in the fact that it was irreducible by a closed technique. Though Williams et al. published closed reduction and casting without internal fixation was possible [10], Miller et al., Richter et al., and Ip and Lui have all advocated the importance of internal fixation in these injuries [5–8]. In this case report, the described dislocation was fixed and could only be reduced with open treatment and furthermore was unstable even after reduction was achieved.

In these injuries, attention must be paid to the often missed talonavicular joint, and the surgeon must be mindful of the skin compromise with delay in diagnosis due to the pressure on the dorsomedial foot. A fixed dislocation of the talonavicular joint can cause extreme skin pressure and put the dorsal skin over the navicular at risk for breakdown. Failure to recognize this urgently could result in a patient being sent home from the emergency room without timely treatment, possibly leading to full thickness skin breakdown.

This case highlights the need to be able to recognize a fixed talonavicular dislocation injury and to avoid complications related to a delay in treatment, missed diagnosis, or skin breakdown.

## 4. Conclusion

Our patient had an irreducible medial swivel dislocation accompanied by concomitant navicular fracture and overlying soft tissue compromise. Closed reduction was unsuccessful, and percutaneous K-wire fixation was needed to stabilize the joint; open fixation of the navicular fracture was added for navicular reconstruction. We recommend internal stabilization as well as navicular reduction with an emphasis placed on concentric talonavicular reduction. It is important to remember that without early recognition, there is a risk of overlying soft tissue breakdown.

We highlight this case not only to bring to light this rare irreducible variant of the swivel dislocation but also to emphasize how timely and technically accurate surgical management can avoid potential complications and ultimately lead to patient satisfaction.
